# Predictors and Prevention of Hallux Valgus Recurrence After Primary Surgical Correction: A Structured Narrative Review

**DOI:** 10.3390/medsci14050537

**Published:** 2026-09-01

**Authors:** Awad Dmour, Ahmed Mansour, Aya Riba, Liliana Savin, Mihaela-Camelia Tîrnovanu, Dragoș-Cristian Popescu, Ștefan-Dragoș Tîrnovanu, Bogdan Puha, Ilie Onu, Norin Forna, Paul-Dan Sirbu, Bianca-Ana Dmour

**Affiliations:** Grigore T. Popa University of Medicine and Pharmacy, 700115 Iasi, Romania; dmour-awad@umfiasi.ro (A.D.); ahmed.o.m326@gmail.com (A.M.); ayastible@gmail.com (A.R.); liliana.savin@umfiasi.ro (L.S.); mihaela.tirnovanu@umfiasi.ro (M.-C.T.); dragos.popescu@umfiasi.ro (D.-C.P.); stefan-dragos.tirnovanu@umfiasi.ro (Ș.-D.T.); ilie.onu@umfiasi.ro (I.O.); norin.forna@umfiasi.ro (N.F.); paul.sirbu@umfiasi.ro (P.-D.S.); bianca-ana.dmour@umfiasi.ro (B.-A.D.)

**Keywords:** hallux valgus, recurrence, bunion surgery, osteotomy, Lapidus procedure, minimally invasive surgery, sesamoid reduction, first-ray instability, metatarsal pronation, recurrence prevention

## Abstract

Hallux valgus recurrence remains one of the most important causes of unsatisfactory outcomes following corrective surgery, with reported rates varying widely because of differences in patient selection, surgical techniques, follow-up duration, and recurrence definitions. Although numerous operative procedures have been described, growing evidence indicates that durable correction depends less on the specific procedure performed than on appropriate patient selection and complete correction of the underlying deformity. A structured literature search was conducted in PubMed from database inception to 4 August 2026 and supplemented by reference-list screening. Adult primary surgical studies reporting recurrence or factors associated with recurrence were eligible when they provided an explicit recurrence definition and at least 12 months of follow-up. Forty candidate primary studies were assessed in full text, of which 30 were included in the core evidence synthesis. Findings were synthesized narratively within predefined domains covering recurrence definitions, preoperative factors, procedure selection, technical correction, postoperative alignment, patient-related factors, and clinical consequences. Particular attention is given to the role of three-dimensional deformity correction, soft-tissue balancing, sesamoid realignment, and radiographic parameters including the hallux valgus angle, intermetatarsal angle, distal metatarsal articular angle, and metatarsal rotation. Contemporary surgical techniques, including Chevron, Scarf, Lapidus, first metatarsophalangeal joint fusion, and minimally invasive procedures, are reviewed with respect to their indications, recurrence mechanisms, and technical considerations. Based on the available evidence, recurrence prevention requires a comprehensive strategy integrating accurate preoperative assessment, procedure selection tailored to the underlying pathology, meticulous surgical technique, and structured postoperative follow-up. Standardized recurrence definitions and greater use of weight-bearing three-dimensional imaging may further improve future research and clinical decision making.

## 1. Introduction

Hallux valgus (HV) is a progressive three-dimensional deformity characterized by lateral deviation of the hallux, medial deviation and pronation of the first metatarsal, and remains one of the most common forefoot pathologies [1] Despite the wide range of surgical techniques available, recurrence continues to represent a significant clinical challenge, affecting up to one in four patients and contributing substantially to postoperative dissatisfaction [2].

Current evidence suggests that durable correction depends not only on achieving adequate three-dimensional correction but also on addressing the underlying biomechanical instability of the first ray. Failure to correct metatarsus primus varus, first metatarsal pronation, and persistent sesamoid malalignment is increasingly recognized as a key contributor to recurrence [3].

Multiple risk factors for recurrence have been described, including increased preoperative HVA and IMA, inadequate postoperative correction, elevated DMAA, and persistent sesamoid malalignment. Their relative contribution remains debated: emerging evidence suggests postoperative alignment, particularly residual HVA and sesamoid position, may be more reliable factors associated with recurrence than preoperative severity [4]. However, interpretation is limited by heterogeneity in how recurrence is defined across studies, ranging from radiographic thresholds to clinical symptom-based criteria.

Accordingly, the aim of this structured narrative review was to critically evaluate the factors associated with recurrent hallux valgus following primary surgical correction in adults. The review examines variation in the definition of recurrence, evaluates preoperative patient and deformity characteristics, assesses the influence of procedure selection and intraoperative correction, and considers postoperative radiographic factors including residual hallux valgus angle, intermetatarsal angle, sesamoid position, distal metatarsal articular angle, and first metatarsal rotation. Particular attention is given to potentially modifiable factors that may improve procedure selection, correction durability, and long-term clinical outcomes, while distinguishing radiographic recurrence from symptomatic recurrence and revision surgery.

## 2. Materials and Methods

### 2.1. Literature Search Strategy

This structured narrative review was conducted to identify and critically synthesize the available evidence regarding hallux valgus recurrence following surgical correction, with emphasis on associated factors, surgical techniques, mechanisms of recurrence, and preventive strategies.

A literature search was performed using PubMed, supplemented by manual screening of the reference lists of relevant systematic reviews and eligible primary studies. Search terms included “hallux valgus,” “recurrence,” “revision surgery,” “Chevron osteotomy,” “Scarf osteotomy,” “Lapidus procedure,” “Akin osteotomy,” “first metatarsophalangeal joint fusion,” “minimally invasive surgery,” “sesamoid reduction,” “metatarsal pronation,” and “first-ray instability.” Priority was given to systematic reviews, meta-analyses, randomized controlled trials, prospective cohort studies, and large retrospective clinical series. Landmark biomechanical studies and relevant reference lists were also reviewed. Owing to the heterogeneity of study designs, recurrence definitions, surgical techniques, and outcome measures, the available evidence was synthesized narratively rather than quantitatively.

A structured literature search was conducted in PubMed from database inception to 4 August 2026. The search combined terms relating to hallux valgus, surgical correction, recurrence, loss of correction, revision surgery, radiographic predictors, sesamoid position, first metatarsal pronation, first-ray instability, osteotomy, and arthrodesis. The electronic search was supplemented by manual screening of the reference lists of relevant systematic reviews and eligible primary studies. No publication-year restriction was applied during study identification. Because this was conducted as a structured narrative review rather than a systematic review, title and abstract screening counts were not prospectively recorded.

### 2.2. Eligibility Criteria and Study Selection

Studies were eligible for the core evidence synthesis when they included adult or predominantly adult patients undergoing primary surgical correction of hallux valgus, reported radiographic or clinical recurrence, loss of correction, or factors associated with recurrence, provided an explicit and reproducible definition of recurrence, and included at least 12 months of postoperative follow-up. Eligible designs included randomized or non-randomized comparative studies, prospective and retrospective cohort studies, and clinically informative case series. Studies were excluded when they consisted of case reports, technical descriptions without recurrence outcomes, conference abstracts, pediatric-only cohorts, revision-only populations, biomechanical or cadaveric investigations, imaging studies without postoperative recurrence outcomes, or studies in which hallux valgus recurrence data could not be separately extracted. Studies with follow-up shorter than 12 months were excluded from the core synthesis. Systematic reviews, technical papers, biomechanical investigations, and rehabilitation studies were retained as contextual sources but were not counted among the included primary studies.

### 2.3. Data Extraction and Evidence Synthesis

Data extracted from eligible primary studies included study design, sample size, surgical procedure, duration of follow-up, definition of recurrence, recurrence frequency, prognostic factors evaluated, statistical approach, and principal findings. Methodological strengths and limitations were evaluated descriptively according to study design, cohort size, completeness and duration of follow-up, clarity of recurrence definition, use of adjusted statistical analyses, and potential selection or attrition bias. Because of substantial heterogeneity in surgical procedures, recurrence thresholds, follow-up duration, radiographic measurements, and statistical reporting, the findings were synthesized narratively rather than quantitatively. Extracted findings were organized into predefined clinical domains comprising recurrence definitions, preoperative predictors, procedure selection, intraoperative and technical factors, postoperative radiographic alignment, patient-related factors, and the clinical consequences of recurrence. This framework was used to derive the thematic structure of the review.

No formal numerical risk-of-bias tool was applied because of the substantial methodological heterogeneity of the included studies. Instead, study design, sample size, duration and completeness of follow-up, clarity of recurrence definition, statistical methodology, and potential sources of selection or attrition bias were considered qualitatively during narrative interpretation.

## 3. Study Selection and Characteristics of the Evidence

**Figure 1:**
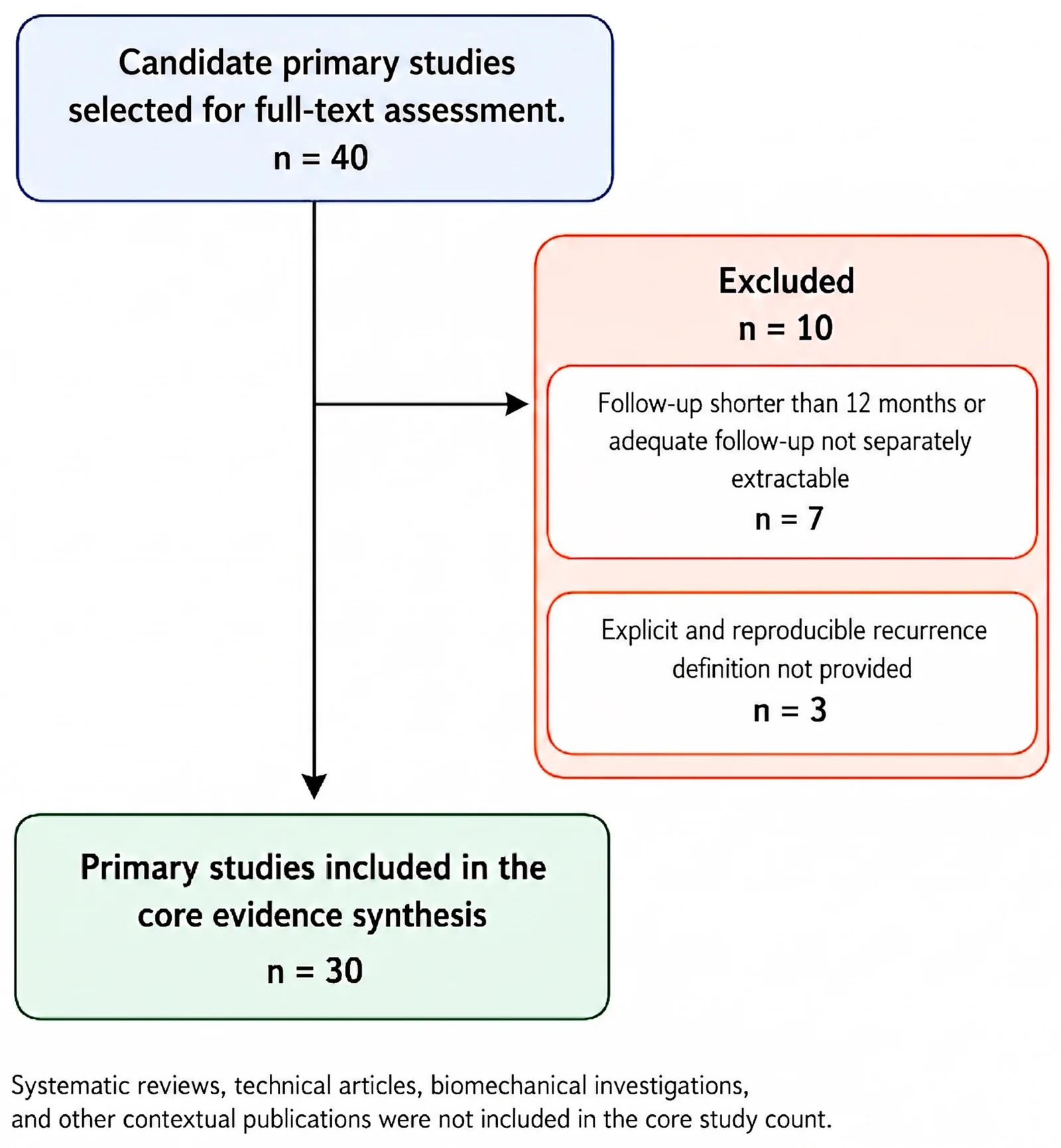
Selection of primary studies included in the core evidence synthesis.

Forty candidate primary studies were assessed in full text. Thirty studies met the predefined eligibility criteria and were included in the core evidence synthesis. Ten studies were excluded: seven because follow-up was shorter than 12 months or results for patients with adequate follow-up could not be separately extracted, and three because an explicit and reproducible definition of recurrence was not provided. The study-selection process is summarized in Figure 1. The characteristics and principal findings of the included studies are presented in Table 1. Contextual systematic reviews, technical articles, biomechanical studies, and rehabilitation publications were used to support interpretation but were not included in the core study count.

## 4. Background: Defining Recurrence and Its Mechanisms

The following definitions were used throughout this review. Radiographic recurrence was defined as the return of the hallux valgus angle (HVA) or intermetatarsal angle (IMA) beyond a predefined threshold—most commonly an HVA > 20°—on postoperative weight-bearing radiographs, irrespective of symptoms. Clinical recurrence referred to the symptomatic return of the deformity associated with pain, footwear-related difficulties, or functional limitations, regardless of whether a specific radiographic threshold was reached. Symptomatic recurrence was defined as patient-reported symptoms attributable to recurrent deformity, which may or may not have fulfilled the radiographic criteria for recurrence. Loss of correction referred to progressive radiographic deterioration relative to the immediate postoperative alignment and may precede clinically significant recurrence. Revision surgery was defined as any secondary surgical procedure performed to address recurrent or residual deformity following primary hallux valgus correction. These definitions were applied consistently throughout the review.

### 4.1. Definition and Heterogeneity of Recurrence

A major limitation in the hallux valgus recurrence literature is the lack of a universally accepted definition. Some define recurrence radiographically, most often HVA > 15° or 20°, while others use clinical symptoms, dissatisfaction, footwear limitation, or revision surgery. This complicates comparison and partly explains the wide range of reported rates [1,2]. Lalevee et al.’s long-term meta-analysis of shaft osteotomies found recurrence varied markedly by threshold: 40% at HVA > 15°, 30% at >20°, 2% at >25° [1].

Radiographic recurrence does not necessarily correspond to symptomatic recurrence: some patients show recurrent angular deformity yet remain satisfied, while others report pain or footwear difficulty despite modest radiographic recurrence. Recurrence should be read as a combined radiographic and clinical outcome rather than a single threshold, since patient-reported outcomes and revision rates may not track a purely radiographic definition (commonly HVA > 20°) [2,3,4]. Therefore, recurrence should be interpreted as a continuum rather than a binary outcome, integrating radiographic findings, patient-reported outcomes, and the need for revision surgery.

Key recurrence parameters include postoperative HVA, residual IMA, sesamoid position, DMAA, first metatarsal rotation, and maintenance of correction over time. Ezzatvar et al. identified preoperative HVA/IMA and postoperative HVA/sesamoid position as significant factors; Park and Lee showed recurrence was associated with immediate postoperative radiographs [1,3].

### 4.2. Preoperative Factors Associated with Hallux Valgus Recurrence

Reported recurrence rates vary considerably by definition, follow-up duration, and procedure, ranging from approximately 10% to over 70%; a pooled prevalence of approximately 25% has been reported [1,2]. Both pre- and postoperative parameters influence this risk: preoperative HVA and IMA showed moderate correlations with recurrence (r = 0.29 and 0.13), while postoperative HVA and sesamoid position showed stronger associations (r = 0.57 and 0.46), underscoring the critical role of adequate intraoperative correction [2]. This underscores careful radiographic assessment and adequate intraoperative correction to reduce relapse risk.

Genetic predisposition may also contribute to recurrence susceptibility. Family and twin studies demonstrate high heritability, while genomic studies have identified several candidate susceptibility loci [5,6,7].

Generalized ligamentous laxity and pes planus have been investigated as contributors to hallux valgus development and recurrence, although evidence remains inconsistent. While clinical studies have not identified ligamentous laxity as an independent factor associated with recurrence, biomechanical data suggest that reduced ligament stiffness may increase deforming forces on the first ray. Hormonal factors, particularly during pregnancy, may also contribute to ligamentous laxity, although their role in postoperative recurrence remains uncertain [8,9].

Pes planus may also alter forefoot biomechanics. While some studies report no significant association with HV severity or recurrence (*p* > 0.05) [10], advanced analyses using deep learning–assisted radiographs demonstrate moderate negative correlations between Meary’s angle and IMA (r = −0.453, *p* < 0.001), with high prevalence of concurrent deformities (53.7% of pes planus feet had HV; 86% of HV feet had pes planus) [11]. Together, laxity and pes planus may predispose to hallux valgus, though causation is unconfirmed and pes planus is best seen as a potential modifier rather than a primary determinant.

Finally, recurrence is consistently linked to a mismatch between procedure and deformity. Patients with medial column instability—first-ray hypermobility, sagittal collapse, or disrupted Meary’s line—are biomechanically “unstable feet” where distal procedures alone can’t maintain correction; such cohorts treated with distal Scarf osteotomy showed up to five-fold higher recurrence [12], while proximal corrections or TMT-1 fusion achieve more durable outcomes [13]. Revision series confirm wrong procedure choice and unaddressed first-ray instability as primary contributors, often needing salvage osteotomies or Lapidus fusion [14].

Collectively, these findings support the concept that hallux valgus recurrence is multifactorial, involving radiographic severity, structural instability, ligamentous characteristics, and procedural mismatch. Comprehensive preoperative assessment of first-ray stability, medial column alignment, rotational deformity, and overall foot biomechanics is essential for appropriate procedure selection and durable correction.

### 4.3. Intraoperative Factors and Technical Determinants of Recurrence

Beyond preoperative deformity characteristics, intraoperative factors play a decisive role in determining the durability of correction. Increasing evidence suggests that recurrence is more often the result of incomplete correction than disease progression alone [2,15].

In this review, undercorrection refers to incomplete restoration of postoperative alignment, typically reflected by residual HVA and/or intermetatarsal angle exceeding the desired correction. This concept should be distinguished from recurrence, which describes progressive return of deformity after initially satisfactory correction. Loss of correction represents the radiographic deterioration occurring over time and may precede clinically significant recurrence.

Residual HVA, inadequate correction of the intermetatarsal angle, and persistent sesamoid malalignment are consistently associated with higher recurrence rates [3,15]. Similarly, uncorrected first metatarsal pronation may lead to progressive loss of alignment despite satisfactory initial radiographic correction [2,3,16].

Soft-tissue balancing is equally important. Inadequate lateral release may prevent complete sesamoid reduction, whereas excessive release without sufficient medial stabilization can result in joint instability or hallux varus [17,18].

Finally, appropriate procedure selection is essential. Distal osteotomies performed in the presence of severe deformity or medial column instability have higher recurrence rates, whereas procedures that address first-ray instability, such as the Lapidus procedure, provide more durable correction when appropriately indicated [12,13].

## 5. Surgical Procedures for Hallux Valgus

The choice of surgical technique is a major determinant of recurrence, but should not be interpreted in isolation. Long-term stability depends on matching the technique to deformity severity, first-ray stability, rotational alignment, sesamoid position, and soft-tissue imbalance. The following procedures are therefore discussed according to their indications, recurrence mechanisms, and technical limitations.

### 5.1. Chevron Osteotomy

Chevron osteotomy is a distal first metatarsal V-shaped osteotomy frequently considered for mild hallux valgus, particularly when deformity severity and first-ray stability are consistent with distal correction alone, where distal correction alone suffices [19]. It is most suitable when first metatarsophalangeal joint congruency is preserved and first-ray hypermobility is minimal.

From a technical perspective, the procedure allows lateral translation of the distal metatarsal fragment to reduce the IMA and realign the hallux, with fixation typically using a screw or K-wire [20,21,22]. For recurrence prevention, the key technical objectives are adequate lateral translation, maintained plantarflexion of the distal fragment, stable fixation, and intraoperative confirmation of sesamoid recentering [20].

Reported recurrence rates after Chevron osteotomy range from 5% to 15%, with higher rates when the preoperative IMA is borderline high, the osteotomy is misoriented, or the sesamoids are not fully recentered. Potential complications include undercorrection, hallux varus from over-resection, avascular necrosis of the metatarsal head, and recurrent deformity from incomplete sesamoid repositioning [21,22].

For this reason, Chevron osteotomy should not be considered a universal procedure, but rather a reliable option for mild deformities with stable medial column mechanics. Failure to restore the relationship between the metatarsal head and sesamoid complex may leave a persistent deforming force, increasing the risk of recurrent valgus drift [23].

### 5.2. Akin Osteotomy

The Akin osteotomy is a medial closing-wedge procedure of the proximal phalanx used to correct hallux valgus interphalangeus, a valgus deformity at the phalangeal level [24]. It is most commonly performed as an adjunct to distal or diaphyseal metatarsal osteotomies, such as Chevron or Scarf, particularly with residual deformity from an increased interphalangeal angle or elevated distal metatarsal articular angle [25].

While the Akin osteotomy improves overall alignment and may enhance cosmetic and radiographic outcomes, it does not address the primary deformity at the level of the first metatarsal. For this reason, its role in preventing recurrence is limited when used in isolation, and it should be considered a complementary procedure rather than a definitive corrective technique [26].

Reported recurrence after combined procedures including an Akin osteotomy varies according to the associated metatarsal procedure, deformity severity, and recurrence definition. In these cases, recurrence is more commonly related to inadequate correction of the intermetatarsal angle, persistent sesamoid malalignment, or unaddressed first-ray instability than to failure of the phalangeal osteotomy itself [27].

Potential complications include hallux varus due to overcorrection, particularly when combined with aggressive soft tissue release [28] as well as lateral cortex fracture found in up to 35.6% of cases and hardware-related irritation reported in approximately 9% [29,30]. Preservation of the lateral cortex and controlled correction are essential to maintain stability and avoid overcorrection.

Overall, the Akin osteotomy should be viewed as an adjunctive procedure that refines alignment and contributes to overall correction, but does not independently prevent recurrence without appropriate correction of the underlying metatarsal deformity [31].

### 5.3. Scarf Osteotomy

The Scarf osteotomy is a diaphyseal Z-shaped first metatarsal procedure designed to correct moderate HV, frequently considered for moderate deformities when first-ray stability is maintained [29,32,33,34,35,36]. It is most suitable for patients with stable first MTP and TMT joints and adequate bone quality, and less appropriate with significant first-ray hypermobility or advanced arthritis.

The technique allows controlled lateral translation of the distal fragment, with stable fixation using screws; although a degree of rotational correction is achievable, complete first metatarsal pronation correction may be more reliably obtained with procedures specifically designed for multiplanar realignment, such as modified Lapidus arthrodesis [33,37]. From a recurrence perspective, key technical objectives include adequate lateral translation, restoration of sesamoid alignment, and preservation of the plantar cortex to prevent troughing and loss of mechanical stability [19,34].

Despite its versatility, the Scarf osteotomy is associated with several failure mechanisms that may contribute to recurrence: insufficient correction of the intermetatarsal angle, incomplete sesamoid reduction, and unaddressed rotational deformity, any of which can cause residual deformity or progressive loss of alignment [34,35,36]. Failure to preserve the plantar cortex can further compromise stability, while overcorrection may lead to hallux varus.

Additional complications include hardware irritation, delayed union, and, rarely, avascular necrosis when vascular supply is compromised [35]. Reported recurrence rates vary considerably depending on follow-up duration and definition: early recurrence ranges from 3.6% to 11.3% [32], whereas long-term follow-up (≥8 to 10 years) demonstrates recurrence rates of up to approximately 40% when a threshold of HVA greater than 15 degrees is applied [1].

These findings suggest that although the Scarf osteotomy provides powerful correction, its long-term durability depends heavily on appropriate patient selection and complete three-dimensional correction, particularly sesamoid alignment and rotational control.

### 5.4. Lapidus Procedure (1st TMT Joint Fusion)

The Lapidus procedure is a fusion of the first tarsometatarsal (TMT) joint that provides durable correction for HV by stabilizing the medial column and first ray [37]. It is primarily indicated for moderate to severe deformities with first-ray hypermobility, medial column instability, or recurrent hallux valgus, and is often appropriate when significant intermetatarsal divergence or instability is present, offering stability often unattainable with distal osteotomies alone [38].

The procedure allows multiplanar correction of the deformity through realignment and stabilization of the first ray, typically achieved using rigid internal fixation with crossed screws or dorsomedial plating [37,39]. From a recurrence-prevention perspective, its major advantage is addressing medial column instability and rotational deformity, both increasingly recognized as important contributors to relapse.

Reported union rates are high (~95%), with clinical recurrence rates around 8% when adequate alignment and stable fixation are achieved [39]. Critical technical considerations include slight plantarflexion of the first ray to reduce transfer metatarsalgia [40], creation of congruent fusion surfaces to minimize nonunion risk [41] and complete correction of metatarsal pronation to avoid residual rotational deformity, which remains an important cause of dissatisfaction and recurrence [42].

Despite its effectiveness, the Lapidus procedure remains technically demanding and carries specific complications, including nonunion, malunion, shortening of the first ray, hardware irritation, and transfer metatarsalgia [37]. In particular, dorsiflexion malunion may compromise first-ray biomechanics and remains one of the leading causes for revision surgery [43].

The Lapidus procedure may be especially advantageous in patients with concomitant pes planus or disrupted Meary’s line, since it stabilizes the first ray in three planes and helps restore the medial longitudinal arch [44]. Comparative analyses show lower recurrence rates with Lapidus–Akin versus Scarf–Akin in medial column instability, supporting procedure selection based on underlying biomechanics.

Overall, the Lapidus procedure represents a durable option for recurrence prevention in appropriately selected patients, particularly when medial column instability is present [45].

### 5.5. First MTP Joint Fusion

First metatarsophalangeal (MTP) joint fusion is an arthrodesis procedure that stabilizes the first MTP joint, primarily indicated for severe or end-stage hallux valgus, recurrent deformity after failed surgery, inflammatory arthropathies, or neuromuscular disorders such as cerebral palsy, where instability and muscle imbalance drive progressive deformity [45,46].

The procedure achieves correction through permanent stabilization of the first MTP joint, with fusion typically performed using crossed screws with or without dorsal plating to maintain compression and alignment [47,48]. From a recurrence-prevention perspective, its major advantage is eliminating the deformity apex and restoring stable alignment when joint degeneration, soft-tissue insufficiency, or neuromuscular imbalance limit the effectiveness of joint-preserving procedures.

Reported complications include malalignment in dorsiflexion, varus, or valgus, nonunion occurring in approximately 5% to 10% of cases, particularly in smokers or patients with poor bone quality, and hardware irritation [47,48]. Malposition of the hallux may significantly affect gait mechanics and footwear tolerance, emphasizing the importance of accurate alignment during fusion.

When fusion is achieved with appropriate positioning and stable fixation, recurrence is uncommon and has been reported in less than 5% of cases [47]. For this reason, first MTP fusion remains one of the most reliable salvage procedures for severe deformity and recurrent hallux valgus.

When residual hallux valgus interphalangeus persists after metatarsal osteotomy or first MTP arthrodesis, an adjunctive Akin osteotomy may be indicated to refine phalangeal alignment, particularly when the interphalangeal angle or DMAA remains elevated after primary correction. Several fixation options are available. Staple fixation provides broad cortical contact and is technically straightforward. Screw fixation delivers axial compression and allows more precise control of wedge closure. K-wire fixation is cost-effective and widely available but requires subsequent removal and carries a risk of pin-tract irritation. Regardless of implant choice, preservation of the lateral cortex is essential to prevent displacement and maintain stability. The Akin osteotomy does not independently prevent hallux valgus recurrence; its corrective effect is confined to the phalangeal level and does not address the underlying first-ray deformity.

### 5.6. Weil Osteotomy (Lesser Toe Correction)

The Weil osteotomy is an oblique distal metatarsal osteotomy used for central metatarsalgia, lesser MTP instability, and lesser toe deformities such as hammer toe, which may coexist with hallux valgus [49,50,51]. The osteotomy is performed parallel to the weight-bearing surface, allowing controlled metatarsal shortening while maintaining a broad bone contact area and relative intrinsic stability [50,52,53]. Fixation is most often a small compression screw, though strategy may vary with bone quality, shortening degree, and associated lesser toe procedures [49].

The most frequent Weil osteotomy complication is floating toe—loss of ground contact during weight-bearing. In a review of 1131 cases, Highlander et al. reported floating toe in 36%, recurrence in 15%, transfer metatarsalgia in 7%, and delayed union/nonunion/malunion together in 3% [49]. Migues et al. and Wagner et al. similarly found floating toe common, without necessarily causing major functional impairment or reducing patient satisfaction scores [54,55].

In hallux valgus surgery, the Weil osteotomy is adjunctive, not primary: it doesn’t correct metatarsus primus varus, first-ray instability, sesamoid malalignment, or the hallux valgus deformity itself, mainly addressing lesser metatarsal overload or toe instability when these coexist. Controlled shortening, preserved sagittal alignment, and avoiding excessive metatarsal elevation minimize postoperative floating toe, stiffness, and transfer symptoms.

### 5.7. Minimally Invasive Surgery (MIS)

Minimally invasive surgery (MIS) includes percutaneous or small-incision osteotomies that realign the first metatarsal and hallux while limiting soft-tissue disruption.

Initially developed for mild deformities, contemporary minimally invasive techniques have demonstrated satisfactory outcomes in selected moderate and severe deformities when performed by experienced surgeons [53,54,56]. Common procedures include percutaneous Chevron osteotomy and third-generation MICA, which correct HVA and IMA through small incisions with stable internal fixation and preserved soft-tissue attachments [57,58].

The proposed advantages of MIS include smaller incisions, reduced soft-tissue trauma, improved cosmetic appearance, lower early postoperative pain, and faster early functional recovery compared with traditional open techniques [53,54,55]. Clinical series have reported significant radiographic and functional improvement after MICA, with low rates of symptomatic recurrence at short to mid-term follow-up [56,57].

However, MIS’s role in preventing recurrence should be interpreted cautiously. Recurrence after MIS relates to the same mechanisms seen after open surgery: undercorrection of the IMA, residual HVA, incomplete sesamoid reduction, and inadequate soft-tissue balancing, plus technique-specific risks during the learning curve such as malrotation and insufficient translation [58]. MIS is not inherently protective against recurrence, but a surgical approach that achieves durable correction with appropriate deformities, meticulous technique, and adequate fixation is protective. Table 2 summarizes the principal surgical procedures used in HV correction, emphasizing their biomechanical rationale, preferred fixation methods, technical pitfalls, dominant failure mechanisms, and reported recurrence or failure.

## 6. Open vs. Minimally Invasive Approaches

Procedure selection provides the structural basis for HV correction, but the surgical approach may also influence soft-tissue trauma, early recovery, complication profile, and technical reproducibility. Open and minimally invasive approaches should therefore be compared not only by radiographic correction, but by their ability to maintain correction over time.

Current evidence suggests minimally invasive correction, particularly third-generation MICA, can achieve outcomes comparable to open distal Chevron or Scarf-type procedures in selected patients, with similar HVA and IMA correction at short-term (<2 years) and mid-term (2–5 years) follow-up (In this review, short-term refers to follow-up of less than 2 years, mid-term to 2–5 years, and long-term to more than 5 years; these definitions are applied consistently throughout and no consistent superiority in patient-reported outcomes [59,60]. Meta-analyses generally support MIS’s clinical validity, though heterogeneity in technique, follow-up, and study design warrants caution; some series favor MIS, others find no clear superiority over open surgery [53,61,62].

MIS offers smaller incisions, less disruption, and faster mobilization, but these don’t make it inherently protective against recurrence. Recurrence after both open and minimally invasive procedures relates mainly to residual HVA, insufficient IMA correction, incomplete sesamoid reduction, persistent metatarsal pronation, and inadequate soft-tissue balancing [56,63]. Learning-curve errors (insufficient translation, malrotation, inadequate fixation) can increase residual deformity; open procedures allow direct visualization but more soft-tissue exposure. Choice should be individualized by deformity severity, first-ray stability, and surgeon experience.

Overall, MIS is a valid alternative to open surgery rather than a universally superior approach: for recurrence prevention, what matters is not incision size but correction adequacy, fixation stability, sesamoid realignment, and balanced soft-tissue forces.

## 7. Technical Determinants of Durable Correction

### 7.1. Soft Tissue Balancing and Capsular Techniques

Soft-tissue management is an often-underestimated determinant of long-term stability. Osteotomies address the bony component, but durable correction also needs balanced periarticular forces; persistent lateral contracture, medial capsular insufficiency, or uncorrected sesamoid displacement can allow recurrent valgus drift even with radiographically adequate bony correction [17,18]. Soft-tissue balancing is thus integral to recurrence prevention, particularly in rigid or severe deformities and cases with persistent sesamoid malalignment after osteotomy.

Lateral soft-tissue release facilitates first metatarsophalangeal joint realignment and sesamoid reduction by releasing contracted lateral structures, including the metatarsosesamoid suspensory ligament, lateral capsule, and adductor hallucis insertion when indicated [17]. Release should be performed selectively based on intraoperative reducibility, residual sesamoid malalignment, and joint stability. Inadequate release may leave persistent deforming forces, whereas excessive release increases the risk of hallux varus and, rarely, avascular necrosis [17,64].

Medial capsular repair is equally important for restoring medial stability. Techniques such as inverted L-shaped capsulotomy, overlapping capsulorrhaphy, and U-shaped capsulorrhaphy have all demonstrated satisfactory results, although current evidence does not support the superiority of any single method [12,65,66].

Adductor hallucis reattachment remains controversial. Although one retrospective study reported improved maintenance of intermetatarsal angle correction after reattachment, the functional benefit was limited, suggesting it may be considered in selected patients rather than as a routine recurrence-prevention strategy [67].

Overall, durable correction depends on restoring balanced soft-tissue forces while avoiding both residual contracture and overcorrection. The objective is balanced correction guided by intraoperative reducibility, sesamoid position, and joint stability.

### 7.2. Sesamoid Realignment

The sesamoid complex plays a central biomechanical role by stabilizing the first metatarsophalangeal joint, improving flexor hallucis brevis function, and contributing to load transmission during gait [17]. In HV, medial displacement of the first metatarsal relative to the sesamoids reflects both transverse-plane deformity and metatarsal pronation.

Postoperative sesamoid malalignment has been associated with recurrence in several observational studies and in previous meta-analytic evidence. However, more recent findings are not entirely consistent, and sesamoid position should not be interpreted as an isolated causal determinant. King et al. reported a graded trend toward higher recurrence with increasing intraoperative tibial sesamoid position, but the association with postoperative HVA recurrence did not reach conventional statistical significance and the study had variable, frequently short follow-up. Sesamoid displacement may therefore be most appropriately regarded as a marker of incomplete three-dimensional correction, incorporating residual metatarsal translation, pronation, and soft-tissue imbalance, rather than as an independent factor associated with recurrence in every surgical context [3,16]. A meta-analysis by Ezzatvar et al. identified postoperative sesamoid position as a stronger factor associated with recurrence than several preoperative parameters, while King et al. found that an intraoperative tibial sesamoid position ≥4 significantly increased recurrence risk [2,68].

Residual sesamoid displacement may indicate incomplete lateral translation, persistent metatarsal pronation, or inadequate soft-tissue balancing. Accordingly, sesamoid reduction should be regarded as a marker of adequate three-dimensional correction rather than an isolated surgical endpoint [69].

Intraoperative assessment of sesamoid reduction depends on the surgical approach: open procedures allow direct visualization and dynamic reducibility checks, confirming tibial sesamoid recentralization. Schneider and Lui both emphasized bony realignment, joint congruity, and sesamoid reduction together [17,18]. Postoperatively, persistent sesamoid displacement should prompt closer follow-up, particularly when accompanied by residual HVA, IMA, or metatarsal pronation, although radiographic recurrence should always be interpreted alongside clinical symptoms [3,70].

### 7.3. Radiographic Factors Associated with Recurrence

Accurate radiographic assessment is essential for identifying patients at risk of recurrence and evaluating correction adequacy. Postoperative alignment is often a stronger predictor of recurrence than the surgical procedure itself, reflecting the combined effects of residual deformity, incomplete correction, persistent sesamoid malalignment, and inadequate restoration of articular orientation.

HVA remains one of the most widely used radiographic parameters, with a postoperative HVA >20° commonly used to define recurrence, although thresholds vary. Hagio et al. found that postoperative HVA at 6 months was independently associated with recurrence after minimally invasive distal linear metatarsal osteotomy, emphasizing the importance of early correction quality [15].

Residual IMA and sesamoid malalignment are also associated with recurrence. Inadequate IMA correction may indicate insufficient lateral translation or unaddressed first-ray instability, whereas persistent lateral sesamoid displacement reflects incomplete correction of the deformity. However, neither parameter should be interpreted in isolation, as their association depends on the overall radiographic and clinical context [3,16].

An increased DMAA may also contribute to recurrence by maintaining lateral hallux deviation despite satisfactory HVA and IMA correction, particularly in moderate to severe deformities [1,71].

**Figure 2:**
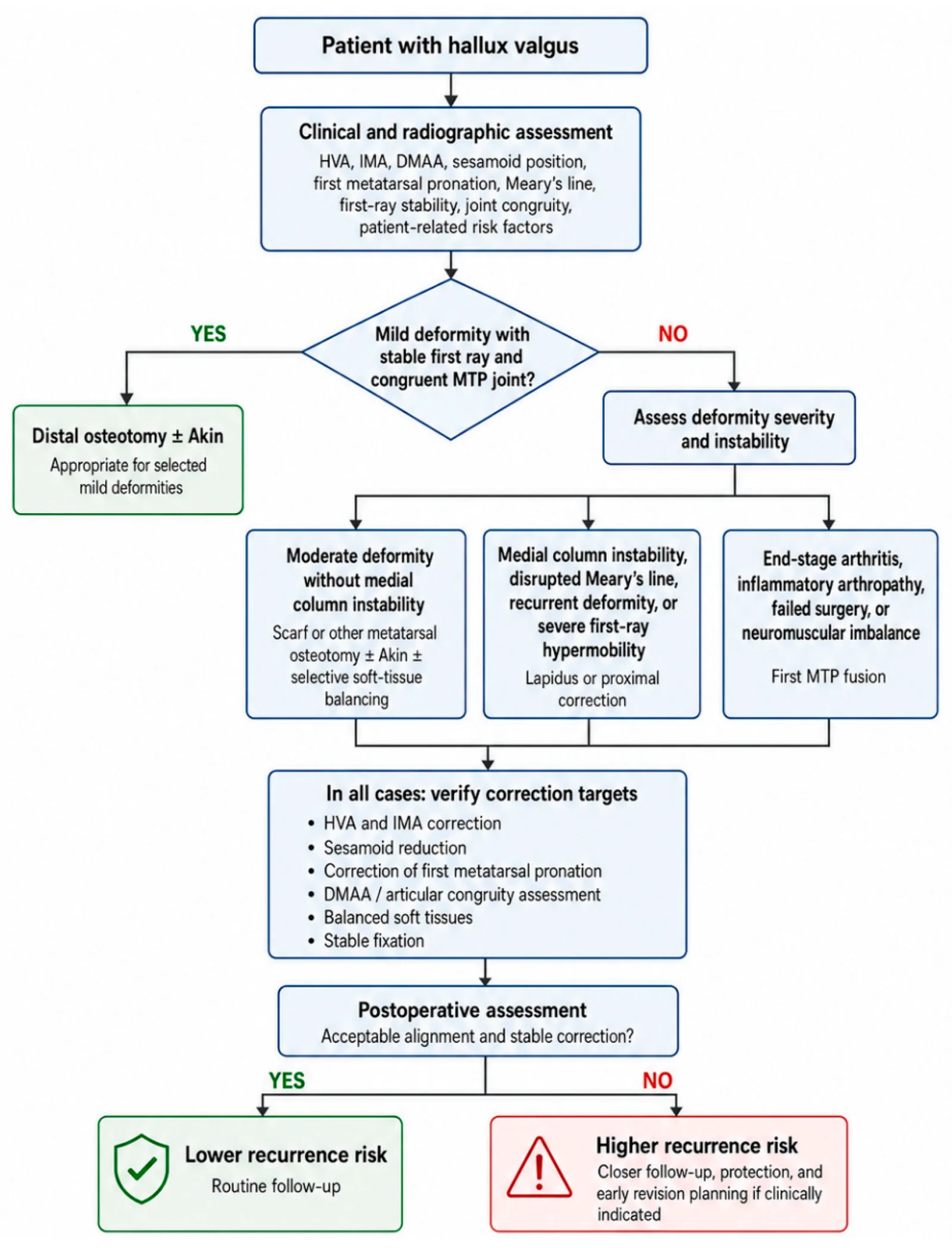
Proposed decision-making algorithm for procedure selection and recurrence prevention in hallux valgus surgery.

Radiographic factors associated with recurrence should be read as an integrated profile: residual HVA, insufficient IMA correction, sesamoid malalignment, elevated DMAA, and uncorrected pronation all signal incomplete correction [2,16,72]. Weight-bearing CT work reinforces metatarsal pronation’s role in this multiplanar deformity, with uncorrected rotation a likely recurrence contributor [73,74]. These mechanisms are summarized in Figure 2; recurrence usually reflects incomplete deformity correction, persistent biomechanical instability, or a mismatch between the chosen procedure and the underlying pathology.

## 8. Postoperative Protocols and Patient-Related Factors

Postoperative management can help maintain intraoperative correction through healing, but is not a substitute for adequate surgical correction. Its role is protective: preserving osteotomy or fusion stability, reducing first-ray overload, and detecting early loss of alignment. Protocols should be tailored to the procedure, fixation stability, bone quality, and deformity severity.

A typical follow-up protocol includes clinical and radiographic assessment at approximately 2 weeks, 6 weeks, 3 months, 6 months, and 12 months, followed by yearly review when clinically indicated. Recent evidence suggests shortened non-weight-bearing may improve early foot function without raising recurrence risk when fixation is stable [75]. Similarly, early mobilization after first MTP arthrodesis appears feasible with rigid fixation, with reported maintenance of union and alignment [76]. This supports a procedure-specific rather than uniform postoperative protocol.

Compliance with postoperative footwear and mobilization also matters. Stiff-soled or rocker-bottom shoes limit stress across osteotomy or fusion sites during healing, though footwear alone hasn’t been proven to prevent recurrence; custom orthoses may help in selected patients but are supportive rather than definitively preventive [75,77,78]. Patient education is essential since premature return to unrestricted footwear may cause pain, loss of correction, or delayed recognition of recurrence [40].

Patient-specific factors also matter. Adolescents and laxity patients have greater medial column mobility and higher recurrence tendency if instability is unaddressed [79,80]. Laxity may increase deforming forces across the first ray [81]. Pes planus increases medial column mobility and alters forefoot load distribution, potentially raising recurrence risk after distal osteotomies that do not stabilize the first ray; first-ray sagittal stability and Meary’s line alignment should therefore be assessed before procedure selection in these patients [12].

Rheumatoid arthritis patients are also high-risk: synovitis, capsular attenuation, joint destruction, and hindfoot deformity can compromise correction, requiring evaluation of forefoot/hindfoot mechanics, disease activity, and fusion strategy alongside the hallux valgus itself [82,83,84].

Reproductive factors may also deserve attention, though their link to postoperative recurrence remains unclear. Parity history may be associated with variation in the first pedal ray angle [85]. Studies in pregnant and parous women report changes in foot anthropometry, including HVA, navicular drop, and foot width/length, suggesting pregnancy-related biomechanical changes may affect forefoot alignment [86]. Parity may thus be an underexplored modifier of hallux valgus progression, but evidence is insufficient to call it an independent factor associated with recurrence.

Overall, postoperative recurrence prevention should be individualized to reflect correction stability, fixation method, the patient’s biological risk profile, and associated deformities such as pes planus, ligamentous laxity, inflammatory arthritis, or lesser ray overload. Close follow-up, structured radiographic surveillance, patient education, and adherence to protected mobilization remain essential to maintain correction and catch early recurrence before it becomes clinically significant.

## 9. Practical Strategy for Recurrence Prevention

Prevention of hallux valgus recurrence should begin by identifying the dominant deformity mechanism rather than selecting a procedure based only on HVA or IMA. Mild deformities with preserved first-ray stability and congruent first MTP alignment may suit distal osteotomies, provided adequate lateral translation, sesamoid reduction, and soft-tissue balance can be achieved. Moderate to severe deformities, disrupted Meary’s line, medial column instability, significant first-ray hypermobility, or recurrent deformity should prompt consideration of more proximal correction or first tarsometatarsal fusion [12,13,51].

Intraoperatively, correction should be assessed as a multiplanar objective. Reduction of HVA and IMA alone is insufficient if sesamoid displacement, metatarsal pronation, DMAA abnormality, or medial capsular insufficiency persists. The surgeon should therefore verify not only angular correction, but also sesamoid recentralization, rotational alignment, articular congruity, and fixation stability [17,74,87,88].

**Figure 3:**
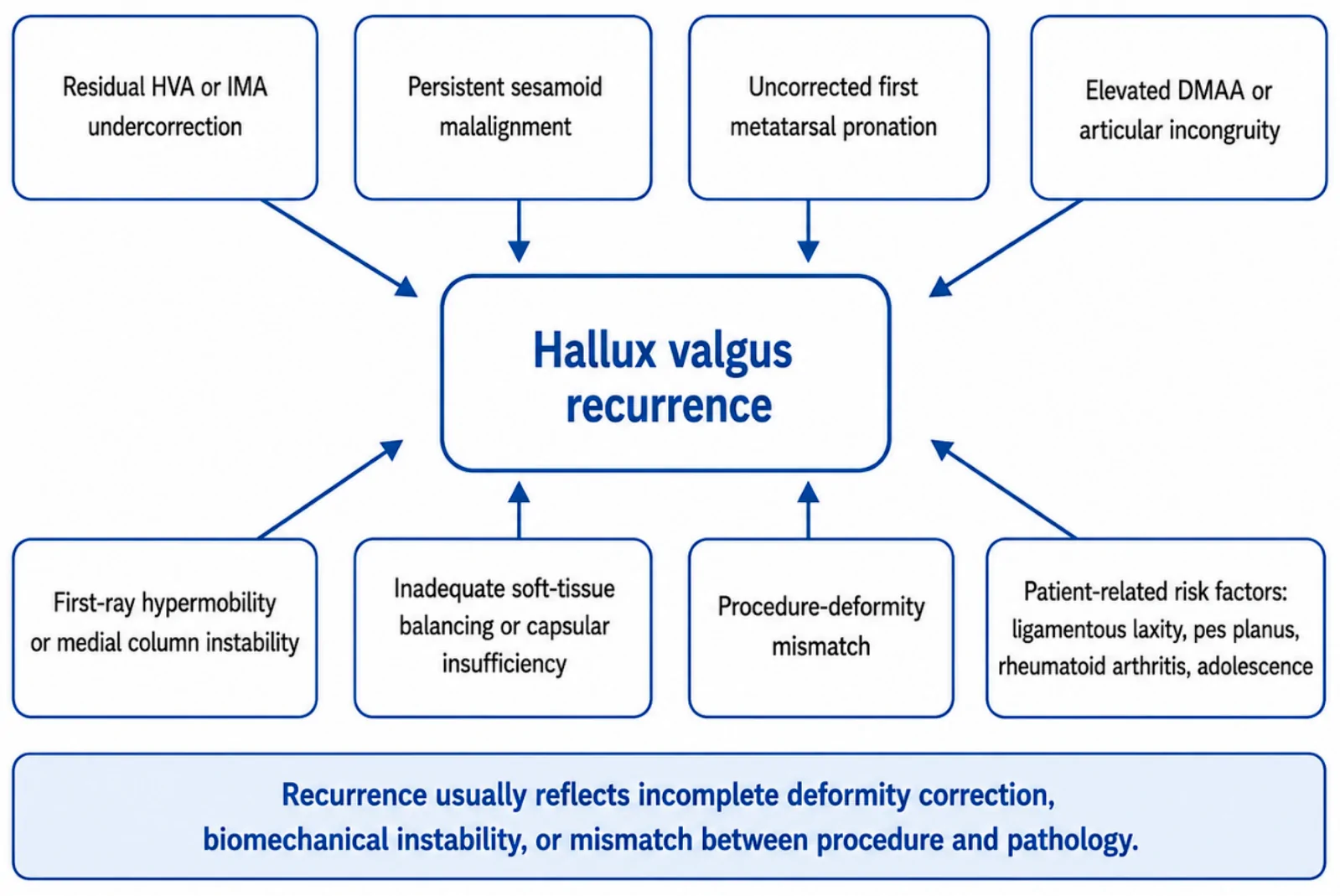
Main mechanisms of hallux valgus recurrence.

Postoperative surveillance should be risk-adapted. High-risk patients—adolescents, those with ligamentous laxity, rheumatoid arthritis, pes planus, medial column instability, or prior recurrence—need closer radiographic follow-up and stricter early protection. Recurrence prevention is thus a continuum: preoperative assessment, procedure selection, technical execution, and postoperative monitoring (Figure 3) [8,75,83].

## 10. Limitations of the Current Evidence

Several limitations should be considered when interpreting the literature on HV recurrence. Recurrence is not defined uniformly: some authors define it radiographically, commonly using a final HVA greater than 20°, while others incorporate symptomatic recurrence, patient dissatisfaction, footwear limitation, or revision surgery. Aiyer et al., for example, defined radiographic recurrence as HVA greater than 20°, illustrating how thresholds vary between studies and directly influence reported recurrence rates [4].

Surgical techniques are also heterogeneous: procedure names such as Chevron, Scarf, Lapidus, or MIS include technical variations that may influence correction durability, particularly for MIS where studies combine different percutaneous technique generations, fixation strategies, and follow-up durations [53,89,90,91].

Many studies are retrospective with selection bias, since severe deformities are often treated with more powerful procedures. This limits direct comparison and makes it hard to separate the effect of procedure, technical execution, surgeon experience, or baseline severity. Comparative studies should be interpreted cautiously unless they account for deformity severity, first-ray instability, and follow-up duration.

Radiographic assessment itself remains imperfect. Conventional two-dimensional radiographs may not fully capture the multiplanar nature of hallux valgus, particularly first metatarsal pronation and sesamoid displacement. Weight-bearing CT has improved understanding of the three-dimensional deformity, and studies on first metatarsal pronation suggest rotational correction may influence recurrence and patient-reported outcomes [74].

Radiographic recurrence doesn’t always correlate with satisfaction, pain, footwear tolerance, or function. Outcome assessment is also complicated by the lack of PROM consensus: Schrier et al. found no consensus on outcome measurement, while Spindler et al. later showed outcome tools remain heterogeneous across studies [92,93]. Future studies should use standardized recurrence definitions, longer follow-up, three-dimensional imaging, and validated patient-reported outcomes to separate radiographic recurrence from clinically meaningful failure.

This review also has methodological limitations. The structured search was restricted to PubMed and reference-list screening, and relevant studies indexed exclusively in other databases may therefore have been missed. Study selection and data extraction were not conducted by two independent reviewers, and no formal numerical risk-of-bias score was applied. The narrative synthesis was intended to accommodate the substantial clinical and methodological heterogeneity of the available literature, but it does not provide pooled estimates of effect. The findings should therefore be interpreted as a structured clinical synthesis rather than as a definitive quantitative comparison of surgical procedures.

## 11. Future Research Directions

Future research should adopt standardized recurrence definitions integrating radiographic and clinical components, adequate long-term follow-up (ideally ≥5 years), weight-bearing CT to quantify three-dimensional deformity, validated patient-reported outcome measures, and prospective comparative studies stratified by deformity severity and first-ray stability.

## 12. Conclusions

Hallux valgus recurrence is a multifactorial outcome influenced by baseline deformity severity, procedure selection, correction of first-ray instability, residual postoperative alignment, sesamoid position, and rotational deformity. The available evidence suggests that the quality of the achieved correction, particularly postoperative HVA and the restoration of multiplanar alignment, may be more informative than the name of the surgical procedure alone. However, recurrence definitions remain inconsistent, and radiographic recurrence does not invariably correspond to pain, dissatisfaction, functional limitation, or revision surgery. Durable correction therefore requires individualized procedure selection, complete three-dimensional correction, balanced soft-tissue management, stable fixation, and follow-up that integrates radiographic and patient-reported outcomes.

## Figures and Tables

**Table 1:** Summary of evidence on risk factors, procedural considerations, and outcome measures associated with hallux valgus recurrence.

Evidence Domain	Factors Evaluated	Overall Finding	Interpretation
Preoperative deformity severity	HVA, IMA, metatarsus adductus	Greater baseline deformity was frequently associated with recurrence	Procedure selection should reflect deformity severity
Postoperative correction	Residual HVA and IMA	Among the most consistent factors associated with recurrence	Early postoperative alignment is clinically important
Sesamoid position	Tibial sesamoid displacement and reduction	Associated with recurrence in several studies, but findings were inconsistent	Best interpreted as a marker of complete three-dimensional correction
First metatarsal rotation	Pronation and round sign	Incomplete rotational correction may increase recurrence risk	Rotational assessment should complement HVA and IMA
Medial column instability	First-ray instability, Meary line disruption, intercuneiform widening	Associated with loss of correction in selected cohorts	May support proximal correction or TMT stabilization
Procedure selection	Distal osteotomy, Scarf, Lapidus, MIS	No procedure was universally superior	Outcomes depend on matching the procedure to the deformity
Follow-up and recurrence definition	HVA thresholds and follow-up duration	Reported recurrence increased with longer follow-up and lower HVA thresholds	Rates cannot be directly compared across studies
Clinical relevance	Pain, satisfaction, revision surgery	Radiographic recurrence did not always correspond to clinical failure	Radiographic and patient-reported outcomes should be reported separately

Abbreviations: HVA, hallux valgus angle; IMA, intermetatarsal angle; MIS, minimally invasive surgery.

**Table 2:** Principal surgical procedures for hallux valgus correction.

Procedure	Biomechanical Rationale and Indication	Preferred Fixation	Primary Technical Pitfall	Most Common Failure Mechanism	Reported Recurrence or Failure Pattern
Chevron osteotomy	Distal V-shaped osteotomy for mild hallux valgus, often appropriate when deformity severity and first-ray stability permit distal correction. Corrects deformity mainly through lateral translation of the metatarsal head.	Headless cannulated compression screw or K-wire fixation	Insufficient lateral translation or misoriented V-shaped osteotomy	Residual IMA, incomplete sesamoid reduction, undercorrection	Approximately 5% to 15%
Akin osteotomy	Medial closing-wedge osteotomy of the proximal phalanx. Corrects hallux valgus interphalangeus and residual phalangeal valgus.	Staple, K-wire, or small screw fixation	Lateral cortical breach or excessive wedge resection	Instability, malunion, hallux varus when overcorrected	Approximately 5% to 12% when used in combined procedures
Scarf osteotomy	Diaphyseal Z-shaped osteotomy for moderate HV. Allows lateral translation, rotation, and shortening when required.	Two compression screws	Troughing, inadequate plantar cortical support, insufficient rotational correction	Loss of correction, residual IMA, persistent sesamoid malalignment	Approximately 3.6% to 11.3% in short to mid-term studies, higher in long-term radiographic follow-up
Lapidus procedure	First TMT arthrodesis for moderate to severe deformity, first-ray hypermobility, medial column instability, or recurrent hallux valgus. Stabilizes the first ray in multiple planes.	Crossed screws, dorsomedial plate, plantar plate, or combined constructs	Incomplete joint preparation, dorsiflexion malposition, inadequate rotational correction	Nonunion, dorsal malunion, transfer metatarsalgia, residual pronation	Approximately 8% in selected series
First MTP fusion	Arthrodesis for severe hallux valgus, end-stage arthritis, inflammatory arthropathy, neuromuscular deformity, or failed previous surgery. Eliminates the deformity apex at the MTP joint.	Dorsal plate with compression screw, crossed screws, or cup-and-cone preparation with plate fixation	Malposition in dorsiflexion, varus, valgus, or rotation	Nonunion, painful malalignment, hardware irritation	Usually <5% recurrence when fusion is achieved
Weil osteotomy	Adjunctive lesser metatarsal shortening osteotomy for metatarsalgia, lesser MTP instability, or transfer overload associated with hallux valgus. Does not correct the primary hallux valgus deformity.	Small compression screw, occasionally K-wire fixation	Excessive shortening, excessive elevation, or sagittal malposition	Floating toe, recurrent metatarsalgia, transfer symptoms, stiffness	Recurrent symptoms approximately 15%; floating toe commonly reported
MIS or MICA	Percutaneous or small-incision distal metatarsal osteotomy, often combined with Akin osteotomy. Corrects HVA and IMA with reduced soft-tissue exposure.	Percutaneous headless compression screws	Insufficient translation, malrotation, incomplete sesamoid reduction, thermal injury during learning curve	Residual deformity, hardware irritation, recurrence from undercorrection	Variable; low in selected short to mid-term MICA series, but dependent on technique and follow-up

Note: Reported recurrence and failure rates vary according to deformity severity, surgical technique, fixation method, follow-up duration, recurrence definition, and whether recurrence is defined radiographically or clinically.

## Data Availability

The original contributions presented in this study are included in the article. Further inquiries can be directed to the corresponding author.
